# TDGN: A text-guided dual-gated network for multimodal sentiment analysis

**DOI:** 10.1371/journal.pone.0349024

**Published:** 2026-05-12

**Authors:** Wenyan Xiao, Lin Zhang, Yangshuyi Xu

**Affiliations:** College of Information Engineering, Shanghai Maritime University, Shanghai, China; Mae Fah Luang University, THAILAND

## Abstract

Multimodal Sentiment Analysis (MSA) aims to interpret emotions by integrating textual, acoustic, and visual information. However, the heterogeneous quality and weak correlation among nonverbal modalities often lead to unstable alignment and ineffective fusion. To address these challenges, we propose a Text-Guided Dual-Gated Network (TDGN) that introduces a hierarchical gating and text-anchored contrastive learning framework. During the alignment phase, a Text-Anchored Gated Attention (TGA) module employs text as a semantic anchor to guide fine-grained alignment between audio and visual modalities while suppressing noise and emphasizing salient emotional cues. For the fusion stage, a Dual-layer Gated Fusion (DGF) module performs intra-modal gating to refine modality-specific features and inter-modal gating to dynamically balance cross-modal contributions. Furthermore, we introduce a Text-Anchored Contrastive Learning (TACL), which constructs contrastive targets based on textual similarity anchors, ensuring the fused features maintain both modal consistency and modal diversity. Extensive experiments on the CMU-MOSI and CMU-MOSEI benchmarks demonstrate that TDGN achieves state-of-the-art performance, achieving higher accuracy and robustness. Ablation studies and visualization further validate the effectiveness of each component.

## 1 Introduction

In recent years, the proliferation of smartphones and social media platforms has led to an explosive increase in multimodal data encompassing text, audio, and video [1,2]. Consequently, sentiment analysis has gradually transcended the traditional single-modal text paradigm, evolving into multimodal sentiment analysis (MSA) that simultaneously leverages multi-source information, including video, audio, and text. Compared to single-modal approaches, MSA provides a more holistic understanding of human affective expression by incorporating auditory and visual cues, thus enriching both perceptual diversity and interpretive accuracy [3].

Despite these advantages, effective MSA remains challenging due to (i) cross-modal alignment and (ii) multimodal fusion [4,5]. These challenges are often coupled by a recurring failure mode: noisy or weakly correlated nonverbal signals may destabilize alignment and subsequently distort fusion, especially when models implicitly assume symmetric reliability across modalities.

Although multimodal cues can be complementary, different modalities often exhibit heterogeneous quality, weak and time-varying correlations, and asymmetric semantic reliability. In particular, audio and visual streams may contain redundancy and environment-dependent noise (e.g., background sounds, pose/illumination changes), whereas text typically conveys more explicit sentiment semantics.

**Fig 1:**
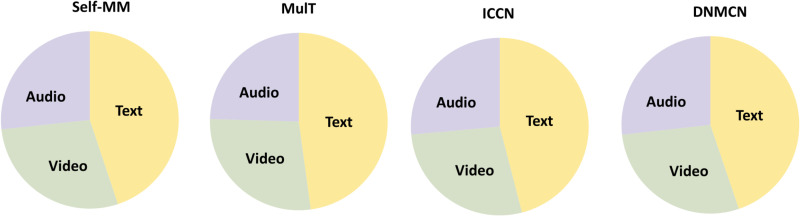
Visualization of the contribution from text, video, and audio modality in sentiment analysis. We evaluate their performance (Acc-2) in the CMU-MOSI dataset with approaches MulT, Self-MM, ICCN, and DNMCN.

To achieve high-quality multimodal feature fusion, the alignment challenge stemming from data heterogeneity must first be addressed. Significant differences in dimensionality and representation formats across modalities mean that simple dimension matching or sequence alignment often overlooks semantic disparities and uneven contributions between modalities, leading to redundant information that degrades overall representation quality. Previous studies attempted to alleviate these issues through cross-modal attention mechanisms or feature concatenation. However, such approaches tend to treat all modalities symmetrically, ignoring the asymmetric contribution of each modality to affect recognition. As shown in Fig 1, which is consistent with findings in prior work [6–8], the text modality demonstrates superior predictive capability compared with the audio and visual modalities. This can be attributed to the linguistic modality’s high semantic density and explicit emotional polarity, whereas audio and visual representations often contain redundant or noisy information that weakens their expressive power and consequently degrades overall MSA performance. Therefore, a text-anchored alignment mechanism that filters noise and highlights semantically salient cues is essential for effective multimodal fusion. Importantly, when noisy audiovisual channels are fed directly into cross-modal attention, spurious correlations may be amplified during alignment and propagate to downstream fusion. This motivates regulating audiovisual features prior to attention-based alignment in our model.

Once semantic alignment has been established, the next critical challenge is to effectively leverage complementary information from all modalities during fusion. With the rise of Transformers, methods like CTFN [9] and MulT [10] introduced cross-modal attention to enhance inter-modal interaction. While these approaches utilize cross-modal self-attention mechanisms to capture inter-modal relationships, they fail to account for the heterogeneity between modalities. Text modalities typically contain richer semantics than video and audio, while video and audio exhibit substantial redundancy and noise. Treating all modalities equally during fusion may lead to suboptimal fusion performance, thereby degrading MSA performance. Experiments from prior studies (TETFN [11], TCAN [12], TeFNA [13] etc.) further validate the contribution of the text modality in MSA, highlighting the importance of text-guided modality fusion. Yet they often suffered from over-assimilation, diminishing the complementary affective cues inherent in non-verbal modalities. Therefore, an effective fusion mechanism should not only leverage the dominant textual semantics, but also adaptively preserve informative modality-specific cues when they are consistent with or complementary to the text.

Despite the progress of existing text-guided and gating-based methods, two limitations remain. First, in many pipelines, modality reweighting is mainly applied after cross-modal interactions have already occurred, which may not sufficiently prevent noisy audiovisual features from affecting the alignment process. Second, many contrastive learning frameworks treat modalities symmetrically, enforcing pairwise similarity without explicitly modeling the semantic dominance of language in sentiment expression. As a result, learned representations may suffer from modality over-suppression or semantic over-assimilation.

Meanwhile, representation-learning-based fusion approaches such as MISA [14] and Self-MM [15] are designed to model both shared and distinct information across modalities, thereby improving MSA performance. However, Self-MM [15] fuses modalities solely by concatenating unimodal representations and projecting them into a low-dimensional space, lacking deep interactive fusion. This motivates exploring fusion designs that can explicitly regulate cross-modal information flow while maintaining both consistency and diversity across modalities.

To overcome these challenges, we propose a Text-Guided Dual-Gated Network (TDGN) model. We introduce a Text-Anchored Gated Attention (TGA) module during the alignment phase, using text as a semantic anchor to guide fine-grained alignment between video and audio modalities. Unlike common text-guided attention that directly attends over raw audiovisual features, TGA applies pre-attention gating to down-weight unreliable audiovisual channels before attention computation, reducing noise propagation during alignment. During the fusion stage, we design a Dual-layer Gated Fusion Module (DGF). Intra-modal gating enhances discriminative features within each modality, while inter-modal gating dynamically adjusts the semantic contribution of multimodal data, achieving global coordination and noise suppression. Regarding training strategies, we introduce Text-Anchored Contrastive Learning (TACL). Moreover, TACL formulates an asymmetric text-anchored contrastive objective to encourage semantic consistency along the textual direction while preventing over-assimilation that may collapse modality-specific affective cues.

Furthermore, unlike contrastive-based fusion models such as ConFEDE, which construct symmetric cross-modal contrastive objectives, TDGN adopts a text-anchored contrastive learning paradigm. By treating text as a semantic reference axis rather than an equal participant, the proposed TACL explicitly enforces semantic consistency along the textual direction while preserving modality-specific diversity. This design complements symmetric contrastive schemes by explicitly modeling the empirical semantic asymmetry in MSA, i.e., using text as a stable reference while retaining nonverbal residual cues. Unlike PS-Mixer, which focuses on disentangling sentiment polarity and intensity at the representation mixing stage, TDGN addresses multimodal heterogeneity earlier by explicitly regulating cross-modal information flow during both alignment and fusion. In TDGN, TGA mainly targets alignment stability by filtering noise before cross-modal attention, whereas DGF focuses on fusion-level modality balancing after alignment; this separation enables more interpretable error correction across stages.

Despite recent progress in MSA, alignment, fusion, and representation regularization are often designed in isolation (e.g., attention for alignment, gating for fusion, and contrastive objectives at the representation level), which limits the ability to jointly address modality imbalance, noisy nonverbal signals, and semantic inconsistency across modalities. The main contributions of this work are summarized as follows:

1. Text-anchored alignment with gated regulation. We propose a Text-Anchored Gated Attention (TGA) module that integrates pre-attention gating with text-guided cross-modal attention, enabling noise-aware alignment between audiovisual modalities and textual semantics.
2. Hierarchical dual-gated multimodal fusion. We design a Dual-layer Gated Fusion (DGF) mechanism that jointly models intra-modal saliency and inter-modal weighting through a hierarchical gating structure, allowing adaptive and balanced multimodal integration.
3. Text-anchored asymmetric contrastive learning. We introduce a Text-Anchored Contrastive Learning (TACL) objective that enforces semantic consistency using textual features as anchors while preserving modality-specific diversity in audiovisual signals.
4. Extensive empirical validation. Experiments on the CMU-MOSI and CMU-MOSEI benchmarks demonstrate that TDGN achieves state-of-the-art performance and robust multimodal representation learning. Additional ablations further quantify the contribution of each module and clarify the functional boundary between alignment and fusion.

## 2 Related work

Sentiment analysis is one of the central research areas in affective computing and deep learning. Over the past decade, sentiment analysis has evolved from unimodal approaches to frameworks that integrate multimodal data. Each modality contributes complementary cues that are semantically interdependent. By jointly modeling these modalities, MSA can infer underlying affective states more accurately than any single modality. In this section, we review recent progress in two fundamental aspects of MSA: multimodal alignment and multimodal feature fusion.

### 2.1 Multimodal alignment

In multimodal data, learning to identify and model correlations among heterogeneous modalities is referred to as learning inter-modal correlations also known as multimodal alignment. The core objective of multimodal alignment is to mitigate inter-modal heterogeneity by mapping diverse modalities onto a unified semantic space, thereby reducing distributional discrepancies and enhancing representational consistency. Effective alignment enables models to better capture complementary information across modalities, forming a prerequisite for high-quality multimodal fusion. This challenge spans multiple granularities – from word and phrase level to segment and aspect level – as evidenced by recent work on multimodal aspect-based sentiment analysis (MABSA) [16,17], which highlights that robust alignment must account for both coarse global correspondence and fine-grained sentiment-related cues under modality noise and content mismatch.

Early alignment methods primarily relied on statistical techniques such as Dynamic Time Warping (DTW) or Canonical Correlation Analysis (CCA), which performed alignment at shallow feature levels. Although these approaches allowed sequence-level or statistical synchronization, they struggled to capture deep semantic associations.

In recent years, with the advancement of deep learning, neural alignment methods have become dominant [18]. Existing alignment approaches can be categorized into implicit and explicit alignment. Implicit alignment does not enforce direct correspondence between modalities; instead, it encourages latent correlation learning through task-driven supervision. Representative work includes MulT [10], which achieves end-to-end alignment using directional cross-modal attention. Similarly, Li et al. [19] introduced a Pseudo-Alignment Algorithm (PAA) that employs one-dimensional convolutions to perform many-to-one alignment between long and short sequences.

In contrast, explicit alignment methods enhance interpretability and semantic consistency by establishing explicit correspondences between modalities at the input or feature layers. For instance, Zhu et al. proposed a DaNet which conducts fine-grained alignment at the aspect level through dual alignment mechanisms for aspect and sentiment perception. Chen et al. [20] introduced a Gated Multimodal Embedding LSTM (GME-LSTM(A)), which explicitly aligns words with corresponding audio/video segments at temporal steps to strengthen temporal-semantic associations. Moreover, Zhu et al. [21] propose the ITINnetwork, which utilizes cross-modal alignment modules to capture semantic correspondences between image regions and words, achieving alignment of visual and linguistic information. Similarly, Li et al. [22] proposed MMTA to explicitly align audio, visual, and textual features at the word level to reduce gaps between heterogeneous modalities. Beyond general MSA, semantic-guided multi-grained alignment has also been explored for MABSA to better associate aspect-related sentiment with cross-modal evidence [17]. However, these methods often overlook semantic asymmetry across modalities, assuming equal contributions to sentiment understanding.

In human communication, language serves as the most direct vehicle for expressing emotions and viewpoints. Textual modalities carry high-density semantic information, enabling clear conveyance of emotional polarity, intensity, and specific targets. In contrast, the emotional signals conveyed by audio and visual modalities are often ambiguous and polysemous, requiring textual context for accurate interpretation. Furthermore, textual sequences possess strict syntactic and semantic structures, whereas feature representations in audio-visual modalities are more prone to inconsistencies and discontinuities [23]. Therefore, using text as the semantic anchor can provide a stable reference for aligning and interpreting non-verbal signals. This view is further supported by related work showing that semantic guidance whether from external knowledge [24] or granularity-aware cross-modal modeling in sarcasm detection [25] consistently improves alignment robustness when inter-modal correlations are weak or unreliable. Based on this understanding, researchers have proposed several text-driven alignment methods recently. Models such as ALMT [26] and TCHFN [27] utilize textual features as queries and audio-visual features as key-value pairs, achieving semantic-level alignment through cross-modal attention mechanisms. Although these methods improved semantic consistency, they remain susceptible to attention dispersion under noisy conditions and lack explicit constraints to enforce alignment stability.

To address these limitations, our proposed TGA module builds upon the “text-as-query” paradigm by integrating a dynamic gating mechanism. The gate functions as a pre-filter that optimizes the quality of audiovisual key–value pairs before attention computation, thereby enhancing the signal-to-noise ratio and improving robustness. This results in semantically coherent and noise-resistant aligned features, providing reliable inputs for subsequent multimodal fusion.

Beyond the sentiment analysis domain, the importance of robust alignment against signal distortion has also been recognized in broader multimedia processing contexts. For instance, handling misalignment under compression artifacts [28] and maintaining stable temporal segment representations under signal perturbations [29] both underscore that effective alignment requires explicit noise regulation – a design principle that motivates our pre-attention gating strategy in TGA.

### 2.2 Multimodal feature fusion

Multimodal fusion plays a crucial role in sentiment analysis, aiming to achieve information complementarity and collaborative modeling across different modalities. Existing methods can be broadly categorized into three types: feature-level fusion, decision-level fusion, and hybrid-level fusion. [1]

Feature-level fusion directly concatenates or projects multimodal representations at the feature layer to form a joint vector for classification [15]. However, it often struggles with modality heterogeneity and redundant information. Decision-level fusion, on the other hand, models each modality independently and combines their outputs through weighted averaging or voting strategies [30]. While this approach enhances overall robustness, it sacrifices fine-grained cross-modal interactions. Hybrid-level fusion integrates both feature-level and decision-level strategies by combining feature-level representations with unimodal predictions, alleviating some limitations of each, yet issues of semantic imbalance and modality redundancy persist.

These conventional strategies generally lack explicit modeling of fine-grained cross-modal interactions, leading to redundant accumulation or excessive reliance on dominant modalities. With the rise of Transformer-based architectures, research focus has gradually shifted toward cross-modal attention–based fine-grained fusion. Models such as MulT [10] and CTFN [9] leverage Transformer structures and modality translation mechanisms to enable deep semantic interactions across modalities. Nevertheless, they fail to fully account for intrinsic modality differences: textual modality typically conveys richer semantic information, while audio and video modalities tend to contain higher redundancy. Treating all modalities equally during fusion may therefore result in suboptimal integration and degraded performance. Beyond attention-based interaction, recent studies incorporate causal inference and uncertainty modeling to reduce spurious cross-modal correlations and improve modality balance, e.g., causality-aware fusion [31], probabilistic causality with cross-modal uncertainty [32], and actual-cause-guided adaptive gradient scaling [33]. Other architectures explore mixture-of-experts and structured relations for heterogeneous modalities, such as polarity-aware MoE fusion [34] and heterogeneous hypergraph attention with counterfactual learning [35].

To address this, TETFN [11] concatenates text-enhanced cross-modal mappings and feeds them into a Transformer encoder for self-attention–driven fusion, achieving more precise sentiment prediction. Similarly, TeFNA [13] introduces a text-centric alignment framework that strengthens the semantic consistency of fused representations. Although these methods highlight the semantic dominance of textual modality, two major limitations remain: (1) the absence of explicit constraints often suppresses weaker modalities, and (2) the tri-modal collaborative modeling is insufficient, leaving room for improvement in robustness and discriminative power. Text-centered designs have also been explored at the sample-interaction level, where cross-sample fusion is used to enhance representation discriminability under noisy modalities [36].

To further enhance modality balance and fusion stability, gating mechanisms have been introduced. MAG-BERT [37] employs a multimodal adaptation gate to perform language-guided semantic modulation; Quan et al. [38] propose a hierarchical gated fusion network to adaptively filter redundant features; Zhang et al. [39] design a two-stage “pre-gating + context calibration” framework to suppress noise propagation. While these approaches regulate modality contributions and mitigate interference, many of them rely mainly on implicit weight learning and provide limited explicit constraints to preserve semantic consistency during fusion.

In parallel, recent studies emphasize joint modeling of modality-invariant and modality-specific features to alleviate modality discrepancy and semantic inconsistency. Related MABSA models also study joint alignment-and-fusion designs, such as multi-level textual–visual alignment and fusion networks [16], which further motivates coupling fine-grained correspondence modeling with fusion. MISA [14] pioneered this direction by designing specific loss functions to disentangle shared and private representations. Building upon this, PS-Mixer [40] introduces a polarity–intensity disentanglement mechanism, performing bidirectional (horizontal and vertical) feature interactions to decompose emotional information into polarity and intensity vectors, thus achieving more efficient and accurate multimodal sentiment analysis. TMBL [41] and MLCL [42] both incorporate cross-modal binding learning to jointly model shared and distinctive representations through bi-modal and tri-modal binding strategies, capturing both common and modality-specific traits. ConFEDE [43] explicitly decomposes each modality into similarity and dissimilarity components and employs text-centered intra- and inter-sample contrastive objectives, thereby enhancing fusion robustness and discriminability at the representation learning stage. These advances have partially mitigated modality imbalance issues in multimodal fusion.

Inspired by these developments, this work proposes a fusion strategy integrating a dual-layer gating mechanism with text-anchored contrastive learning. By treating text as a semantic reference in contrastive learning, the proposed model guides audio and visual modalities to maintain consistency while preserving modality-specific diversity. Meanwhile, the dual-layer gating mechanism dynamically balances modality contributions and explicitly models cross-modal complementarity. Together, these components enable synergistic and complementary multimodal interactions, substantially improving the overall robustness and discriminative capability of the fusion process. From a representation-learning perspective, autoencoder-based hashing for image retrieval provides a reference for compressing representations while maintaining discriminability, which is conceptually related to learning compact and robust multimodal embeddings [44]. Moreover, multi-stage dilated temporal convolution networks for action segmentation highlight an alternative to Transformer-style temporal modeling and may inspire sequential feature extraction designs for long, noisy audiovisual streams [45].

Accordingly, TDGN integrates text-guided dual-layer gated fusion with text-anchored contrastive regularization to achieve robust and semantically consistent multimodal sentiment modeling.

### 2.3 Differences from prior work

Although text-guided alignment, gating-based fusion, and contrastive learning have been extensively studied in multimodal sentiment analysis (MSA), TDGN is not a mere composition of these techniques. Instead, it introduces a unified, asymmetry-driven architectural paradigm that fundamentally reorganizes their interaction.

Alignment Strategy (vs. ALMT and MAG-BERT). ALMT [26] employs text as the query in cross-modal attention, directly attending to raw audiovisual features, which makes alignment susceptible to modality-specific noise. MAG-BERT [37] performs language-conditioned gating after multimodal feature extraction, focusing on semantic adjustment rather than noise suppression. In contrast, the proposed Text-Anchored Gated Attention (TGA) applies gating prior to cross-modal attention, explicitly filtering unreliable audiovisual signals before interaction. This pre-attention mechanism enhances the signal-to-noise ratio and mitigates spurious correlations during alignment.

Contrastive Learning–Gating Coupling (vs. ConFEDE). ConFEDE [43] adopts text-centered contrastive learning as an independent representation learning objective. In TDGN, Text-Anchored Contrastive Learning (TACL) is structurally integrated with the Dual-layer Gated Fusion (DGF) module, directly regulating both intra- and inter-modal gating behaviors. This coupling constrains fusion dynamics at inference time, preventing over-integration of noisy nonverbal cues, rather than merely shaping representations during training.

Disentanglement and Feature Mixing (vs. MISA and PS-Mixer). MISA [14] enforces similarity and orthogonality constraints symmetrically across modalities, implicitly assuming equal reliability. PS-Mixer [40] focuses on polarity–intensity disentanglement during feature mixing but does not address upstream cross-modal heterogeneity. TDGN resolves these limitations through a unified asymmetric design: text serves as the sole semantic anchor, defining the invariant shared subspace, while audiovisual modalities are treated as complementary residuals. Furthermore, heterogeneity is handled in two stages-TGA performs pre-alignment noise suppression, and DGF conducts post-alignment modality balancing-thereby decoupling noise filtering from feature fusion.

## 3 Method

**Fig 2:**
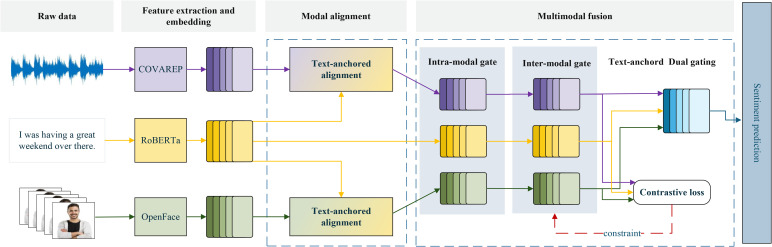
Overall architecture of the TDGN. Different colors represent different modalities: yellow for text modality, purple for audio modality, and green for visual modality.

This section provides a detailed description of the proposed TDGN. As shown in Fig 2, TDGN consists of three primary components: feature extraction, cross-modal alignment, and cross-modal fusion. Section 3.1 introduces the feature extraction module, Section 3.2 details the implementation of cross-modal alignment, and Section 3.3 describes the fusion of aligned modalities.

### 3.1 Feature extraction

The feature extraction module serves as the foundation of the entire architecture, responsible for extracting unified dimensional feature representations from raw data across text, audio, and video modalities. The feature extraction module comprises two primary stages: feature extraction and feature embedding. During feature extraction, we employ pre-trained models-RoBERTa [46], OpenFace [47], and COVAREP [48] to process text, video, and audio data respectively. In the feature embedding stage, we project features from all three modalities onto a common dimensionality to achieve higher-quality information and lower computational complexity.

#### 3.1.1 Text feature.

Text features are extracted using a pre-trained model RoBERTa. The textual input is first tokenized and represented as input_ids and attention_mask, which are then passed through the model to obtain the last hidden-layer outputs containing rich semantic information. Formally, these hidden states retain rich textual semantic information, providing effective contextual feature representations for subsequent sentiment analysis. The representations of raw sentences $S_{t}\in {\mathbb{R}}^{T\times d_{m}}$ are mapped into low-dimensional vectors $U_{t}\in {\mathbb{R}}^{d_{h}}$ according to the following equation:


$$U=\text{RoBERTa}(S_{t},\theta_{t})$$ (1)

where $\theta_{t}$ represents the parameters of the RoBERTa model. Specifically, we use the last hidden-layer outputs as token-level representations; for fixed-length representation, attention pooling is applied to aggregate the sequence.

#### 3.1.2 Audio feature.

For audio, we use the COVAREP toolkit to extract frame-level acoustic features. The general workflow of COVAREP for audio processing is as follows: after inputting the raw audio signal, the COVAREP toolkit sequentially extracts dozens of acoustic features, including peakSlope, QQQ, and MDQ. These features are then stacked and integrated to form the final feature representation *S*_*a*_. To model long-range dependencies and temporal context, a Transformer-based audio encoder is employed. The audio features are first mapped to the Transformer’s hidden dimension via an input projection layer. Learned positional encodings are then added to preserve temporal information.


$$H_{a}=\text{TransformerEncoder}(\text{LinearProj}(S_{a})+\text{PositionEncoding})$$ (2)

$$U_{a}=\text{AttentionPooling}(H_{a})$$ (3)

where $S_{a}\in {\mathbb{R}}^{T\times d_{i}}$ denotes the input audio feature sequence, and $U_{a}\in {\mathbb{R}}^{d_{h}}$ denotes the encoded fixed dimensional representation. Attention pooling aggregates frame-level information into a fixed-length vector that captures prosodic and emotional cues while reducing noise sensitivity.

#### 3.1.3 Visual feature.

Visual features are extracted using the OpenFace toolkit. OpenFace first detects facial regions via the HOG algorithm, then locates facial landmarks with the ERT algorithm, and finally generates facial embeddings with a convolutional neural network. Subsequently, the model employs a spatio-temporal Transformer to decompose video information into temporal and spatial dimensions, allowing temporal and spatial features to be modeled separately. The temporal layer first models the entire video sequence to capture long-range dependencies between frames, while the spatial layer treats each temporal step’s features as independent samples to model interactions across different feature channels. Spatio-temporal information is ultimately integrated through residual connections and layer normalization. To further preserve inter-frame temporal relationships, a learnable temporal position encoding is introduced to maintain the sequential order within the video frame sequence effectively. The implementation formula for the spatio-temporal transformer is as follows:


$$H=\text{TemporalTransformer}(X_{v}+E_{\text{temp}})$$ (4)

$$U_{v}=\text{Pooling}(\text{LayerNorm}(\text{SpatialTransformer}(H)+H))$$ (5)

where $X_{v}\in {\mathbb{R}}^{T\times d_{v}}$ denotes the input video feature sequence, *E*_temp_ represents the temporal position encoding, and $U_{v}\in {\mathbb{R}}^{d_{h}}$ is the final output video representation. The combination of temporal and spatial encoders enables the model to capture dynamic facial expressions and motion cues critical to emotion recognition.

The OpenFace and COVAREP features are pre-extracted and fixed during training; only the projection layers and downstream encoders are trained end-to-end. All three modalities are projected to a unified hidden dimension of $d_{h}=128$, balancing representational capacity and computational efficiency. We acknowledge that the use of fixed pre-extracted audio-visual features may limit the model’s sensitivity to subtle temporal dynamics such as micro-expressions and prosodic variation; this limitation is further discussed in Section 5.

### 3.2 Feature alignment

Cross-modal alignment aims to establish a semantically consistent space among the three modalities. Unlike conventional cross-modal attention frameworks that directly compute attention between modalities, the proposed TGA module introduces an element-level pre-attention gating mechanism that explicitly regulates individual feature channels before attention computation. This design prevents noisy audiovisual signals from dominating the alignment process and improves the stability of cross-modal correspondence.

**Fig 3:**
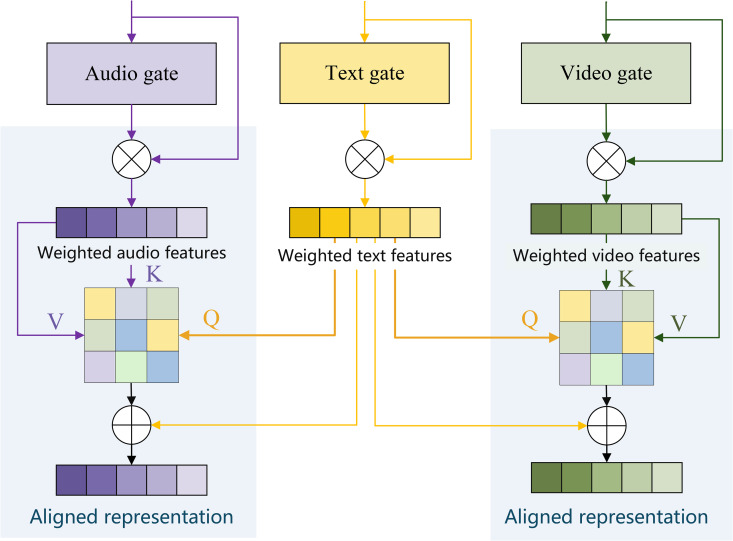
The architecture of the proposed Text-Anchored Alignment module. Text, audio, and video features are first filtered through modality-specific gating networks to highlight semantically relevant information and suppress noise. Subsequently, text features act as queries (Q) in cross-modal attention, while gated audio and video features serve as keys (K) and values (V). This design enforces semantic alignment across modalities under text guidance, yielding noise-robust aligned representations.


As illustrated in Fig 3, TGA first applies a linear transformation and Sigmoid activation to each modal input $h_{m}\left(m\in \{t,v,a\}\right)$, generating a gated weight vector *g*_*m*_, and then performs channel-wise scaling:

$$g_{m}=\sigma (W_{m}h_{m}+b_{m}),{\tilde{h}}_{m}=g_{m}⊙h_{m}$$ (6)

where σ denotes the Sigmoid function, and *W*_*m*_, *b*_*m*_ are learnable parameters. The gating structure attenuates irrelevant dimensions during the forward pass while amplifying key semantic channels. Simultaneously, it imposes constraints on gradient updates during backpropagation, suppressing gradient oscillations in noisy channels to stabilize alignment optimization.

Subsequently, the TGA module leverages the clear semantic information provided by the text, allowing it to act as a filter to actively retrieve the most emotion-relevant segments from noisy audio and video sequences while suppressing irrelevant information. Specifically, the TGA module uses the text features as the query, with the gated audio and video features serving as the key and value respectively. It achieves semantic alignment through cross-modal attention calculations:


$$A_{t\to m}=\text{softmax}\left(\frac{(W_{Q}h_{t})(W_{K}{\tilde{h}}_{m}{)}^{⊤}}{\sqrt{d_{k}}}\right),\;m\in \{v,a\}$$ (7)

Among these, *W*_*Q*_, *W*_*K*_ represent the query-key mapping matrices, respectively, while *d*_*k*_ denotes the scaling factor. By computing the similarity between text modality and each of the audio and visual modalities, the model establishes a semantic correspondence between text and audio/video representations.

Based on the attention weights $A_{t\to m}$ the model performs weighted aggregation on the gated modal features, yielding a semantically enhanced alignment representation:


$$h_{m}^{\text{aligned}}=A_{t\to m}(W_{V}{\tilde{h}}_{m}),\;m\in \{v,a\}$$ (8)

Here, *W*_*V*_ denotes the value mapping matrix, and $h_{m}^{\text{aligned}}$ represents cross-modal aligned features consistent with the text semantics. Through the mechanism, the text feature *h*_*t*_ serves as a semantic anchor. During the alignment phase, it actively retrieves emotion-related segments from the audio and video modalities that align with its semantic content while effectively suppressing irrelevant signals. Concurrently, the pre-processed gated filtering suppresses noise dimensions during the forward pass and regulates gradient flow during backpropagation, ensuring the model exhibits higher stability and robustness in both training and inference stages. The aligned representation, denoted as *H*_aligned_, produced by this module serves as input to the subsequent dual-layer gated fusion module, laying the semantic foundation for effective multimodal information collaboration.

### 3.3 Modality fusion

**Fig 4:**
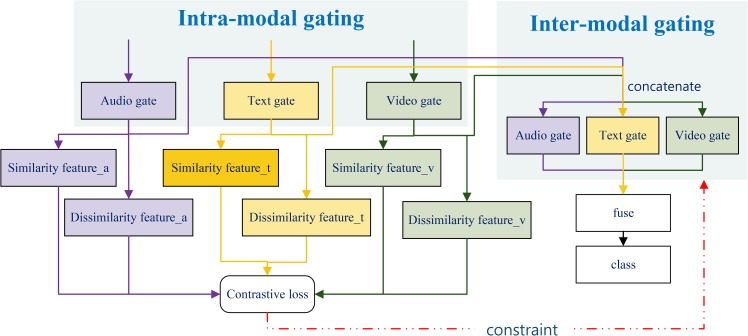
Architecture of the Dual-layer Gated Fusion Module. The module integrates intra-modal gating for contrastive feature separation and inter-modal gating for weighted feature integration. The contrastive loss objective enhances the discriminative power of the individual modalities, while the inter-modal module facilitates global information fusion for final classification. A dashed red line indicates the constraint feedback used to regularize the entire fusion process.

In multimodal sentiment analysis, effectively integrating different modalities is crucial for enhancing overall performance. We propose a dual-layer gated fusion module that combines text-guided contrastive learning. It dynamically learns the importance of each modality from semantic cues and adaptively adjusts the fusion weights. Simultaneously, it uses text as semantic anchors to enhance cross-modal semantic consistency through contrastive learning. This module comprises intra-modal gating and inter-modal gating. The former highlights key information across modalities, while the latter dynamically balances modal contributions, enabling progressive fusion from local features to global semantics. The architecture of the dual-layer gated fusion module is illustrated in Fig 4.

(1) Intra-modal Gating. The intra-modal gating operation follows the same formulation as Eq. 6, where an element-wise Sigmoid gating mechanism is applied to each modality to control information flow. Specifically, it serves a feature refinement role—enhancing discriminative channels within each modality and acting as a noise filter at the modality-specific level, thereby suppressing redundant dimensions while emphasizing salient modality-specific features.
(2) Inter-modal Gating

In contrast to intra-modal gating, inter-modal gating operates at the global fusion level. Modal gating operates at the element level and adaptively assigns weights based on semantic consistency within the global context. By concatenating features from each modality and applying Sigmoid activation, the interaction weights between modalities are obtained:


$$\left[\alpha_{t},\alpha_{v},\alpha_{a}]=\sigma (W_{\text{inter}}[{\tilde{h}}_{t};{\tilde{h}}_{v};{\tilde{h}}_{a}]+b_{\text{inter}}\right)$$ (9)

Feature fusion is jointly determined by modally weighted features and inter-modal weights:


$$h_{\text{fused}}=\alpha_{t}{\tilde{h}}_{t}+\alpha_{v}{\tilde{h}}_{v}+\alpha_{a}{\tilde{h}}_{a}$$ (10)

Here, $\alpha_{m}\in [0,1]$ denotes the dynamic weight between modalities. When text and video share consistent semantics, their interaction weights $\alpha_{t},\alpha_{v}$ are simultaneously amplified; when semantic conflicts arise, the gate automatically learns the more reliable modality and suppresses the influence of the other. This layer achieves global balance and dynamic coordination among text, audio, and video, thereby maintaining robustness under multimodal imbalance conditions. To prevent trivial solutions where dominant weights are always assigned to the text modality, the gating mechanism is jointly constrained by multi-modal supervision and contrastive objectives. This encourages balanced modality utilization rather than collapsing to a single dominant source.

(3) Text-Anchored Contrastive Learning

To prevent over-assimilation induced by strong textual guidance while preserving the distinctive cues of non-textual modalities, we introduce a lightweight feature decoupling mechanism combined with text-anchored contrastive learning at the fusion stage. Rather than performing full shared–private representation decomposition, the proposed strategy selectively separates text-consistent semantic components from modality-specific residual information. This design enables semantic regularization guided by language without collapsing complementary affective cues carried by audio and visual modalities.

To mitigate multimodal semantic overlap, each modality’s representation is decoupled into two components: (1) similarity features, which capture semantic information aligned with textual expressions and serve as anchors for text-guided contrastive supervision; and (2) dissimilarity features, which retain modality-specific residual cues and function as complementary sentiment signals outside the contrastive constraint.

Specifically, we decompose the gated representations of each modality into similarity features aligned with the textual direction and orthogonal dissimilarity features. We first define the average direction for audiovisual modalities:


$$r=\frac{{\hat{h}}_{v}+{\hat{h}}_{a}}{\left\|{\hat{h}}_{v}+{\hat{h}}_{a}\right\|}$$ (11)

Subsequently, the textual features are decomposed into similarity features and dissimilarity features.


$$h_{t}^{\text{sim}}=\left\langle {\hat{h}}_{t},r\right\rangle r,\;h_{t}^{\text{dis}}={\hat{h}}_{t}-h_{t}^{\text{sim}}$$ (12)

Among these, ${\hat{h}}_{t}$, ${\hat{h}}_{v}$, ${\hat{h}}_{a}$ represent the normalized text, video, and audio features, respectively. Since both r and ${\hat{h}}_{v}$ are L2-normalized, the projection coefficient $\left\langle {\hat{h}}_{t},r\right\rangle$ is bounded within [−1, 1], which precludes numerical degradation and ensures that the orthogonal projection remains numerically stable in high-dimensional spaces. This decomposition ensures that each modality simultaneously preserves shared semantics consistent with the text while retaining its own unique complementary information.

During the fusion stage, text-anchored contrastive constraints are introduced to enhance cross-modal semantic consistency. This loss function uses $h_{t}^{\text{sim}}$ as the anchor, maximizes consistency between text similarity features and video/audio similarity features ($h_{v}^{\text{sim}},h_{a}^{\text{sim}}$) while minimizing similarity with all dissimilarity features ($h_{t}^{\text{dis}},h_{v}^{\text{dis}},h_{a}^{\text{dis}}$) and cross-sample features. The loss function is defined as:


$${ℒ}_{\text{con}}=-\frac{1}{B}\sum_{i=1}^{B}\log\frac{\sum_{p\in P_{i}}\exp\left(\text{sim}\left(h_{i}^{\text{sim}},p\right)/\tau \right)}{\sum_{c\in C_{i}}\exp\left(\text{sim}\left(h_{i}^{\text{sim}},c\right)/\tau \right)}$$ (13)

where *B* denotes the batch size, τ represents the temperature parameter, *P*_*i*_ denotes the positive sample set for the *i*^*th*^ anchor (including $h_{v}^{sim},h_{a}^{sim}$), and *C*_*i*_ denotes the candidate set (encompassing all positive and negative samples). Compared to contrastive learning using only a single positive sample, the multi-positive sample mechanism adopted in this article (simultaneously considering text–video and text–audio) constructs a “semantic consensus” constraint. This effectively enhances the stability of gating learning and the clarity of semantic guidance, thereby promoting the adaptive convergence of fusion weights toward a shared semantic space. This loss guides the model during backpropagation to strengthen feature components aligned with semantic consistency, making inter-modal gating weight allocation more inclined toward affect expressions consistent with textual semantics.

In summary, the proposed dual-layer gated structure effectively highlights discriminative features within each modality while achieving adaptive weight balancing across modalities. Furthermore, text-anchored contrastive learning ensures consistency in semantic alignment direction. The resulting *h*_*fused*_ feature combines robustness and discriminative power, providing high-quality feature representations for sentiment classification tasks.

### 3.4 Objective functions

To address the multi-grained nature of multimodal sentiment analysis, we first clarify the model’s output and evaluation protocol. Specifically, the model predicts a single continuous sentiment score $\hat{y}\in \mathbb{R}$ at the final output layer. This continuous prediction seamlessly supports both evaluation paradigms without ambiguity: $\hat{y}$ is evaluated directly for regression metrics (MAE and Corr), and mapped to discrete categories via standard thresholds (e.g., $\hat{y}>0$ for positive, $\hat{y}\leq 0$ for negative, and rounded to the nearest integer for 7-class) to compute classification metrics (Acc-2, Acc-7, F1). Drawing upon the research in PS-Mixer [40], this study jointly optimizes three complementary loss functions during training: regression loss (${ℒ}_{\text{reg}}$), polarity loss (${ℒ}_{\text{polar}}$) and intensity loss (${ℒ}_{\text{strength}}$). The overall optimization objective is defined as follows:


$${ℒ}_{\text{total}}=w_{1}\cdot {ℒ}_{\text{reg}}+w_{2}\cdot {ℒ}_{\text{polar}}+w_{3}\cdot {ℒ}_{\text{strength}}$$ (14)

where *w*_1_, *w*_2_, *w*_3_ are hyperparameters used to balance the contributions of polarity loss and intensity loss in the overall objective function. Regression Loss (${ℒ}_{\text{reg}}$): Measures the discrepancy between the predicted continuous sentiment score and the ground-truth label using Mean Squared Error (MSE):


$${ℒ}_{\text{reg}}=\text{MSE}(\hat{y},y)=\frac{1}{N}\sum_{i=1}^{N}({\hat{y}}_{i}-y_{i}{)}^{2}$$ (15)

Polarity Direction Loss(${ℒ}_{\text{polar}}$) is a directional loss based on cosine similarity that explicitly constrains the correctness of sentiment direction (positive/negative). This loss optimizes the consistency between the predicted polarity vector and the true direction vector:


$${ℒ}_{\text{polar}}={ℒ}_{\text{polar}}({𝐯}_{\text{polar}},y,\hat{y})$$ (16)

Strength Loss(${ℒ}_{\text{strength}}$): A correlation-based loss function that accurately models sentiment intensity, optimizing the correlation between ‘scale‘ and the true label to ensure sentiment strength accuracy:


$${ℒ}_{\text{strength}}={ℒ}_{\text{strength}}(s,y)$$ (17)

This joint optimization strategy effectively bridges the gap between regression and classification goals. By simultaneously optimizing the exact continuous score, its directional polarity, and its absolute intensity, the model captures a more comprehensive representation of multimodal sentiment, thereby ensuring high fidelity in continuous predictions and robustness in discrete classification.

## 4 Experiment

This section describes the experimental setup and analysis of results. We evaluate the model’s performance on two public datasets, detailing the datasets used, baseline models, and experimental outcomes.

### 4.1 Dataset

Our experiments were conducted on two publicly available MSA datasets, CMU-MOSI and CMU-MOSEI. The composition of the two datasets is shown in Table 1.

**Table 1:** Dataset split.

Dataset	Train	Valid	Test	All
CMU-MOSI	1284	229	686	2199
CMU-MOSEI	16326	1871	4659	22856

The CMU-MOSI [49] dataset comprises 93 videos collected from YouTube, featuring speakers commenting on specific topics. Each video segment is manually labeled on a scale of [−3, 3], representing emotional intensity ranging from strongly negative to strongly positive. The dataset contains 26,295 words from 2,199 opinion-based video utterances, annotated with emotional intensity labels ranging from −3 (strongly negative) to +3 (strongly positive).

The CMU-MOSEI [50] dataset is currently the largest multimodal sentiment analysis dataset. It covers 250 distinct topics from 1,000 unique speakers, comprising 23,353 labeled video segments extracted from 2,928 videos. Each segment includes sentiment intensity labels alongside six emotion labels such as happiness and sadness.

### 4.2 Evaluation metrics

For the CMU-MOSI and CMU-MOSEI datasets, sentiment is categorized into seven intensity levels: [−3, −2) denotes highly negative, [−2, −1) denotes negative, [−1, 0) denotes weakly negative, [0] denotes neutral, (0, 1] denotes weakly positive, (1, 2] denotes positive, and (2, 3] denotes highly positive. We established two evaluation tasks: regression and classification. For the regression task, we utilize Mean Absolute Error (MAE) and Pearson Correlation Coefficient (Corr) as evaluation metrics. For the classification task, we adopt 7-level accuracy (Acc-7), 2-level accuracy (Acc-2), and F1 score (F1) as evaluation metrics. Acc-2 and F1 are evaluated in two scenarios: neg/non-neg and neg/pos. Corr is defined as the quotient of covariance and standard deviation between two variables, while MAE represents the average absolute error between actual and predicted values. Higher values indicate better performance, except for MAE.

### 4.3 Experimental parameters

The model is trained using the Adam optimizer for a maximum of 100 epochs, with early stopping applied when validation MAE shows no improvement for 10 consecutive epochs. All experiments are conducted under 5 fixed random seeds (55654, 42, 123, 2025, and 74), and results are reported as mean ± standard deviation across runs. The hierarchical learning rate strategy sets the RoBERTa encoder at 5 × 10^−5^ and all remaining module parameters at 1 × 10^−3^. Batch sizes are set to 128 for CMU-MOSI and 64 for CMU-MOSEI.

Input features from RoBERTa ($d_{m}=768$), COVAREP ($d_{a}=74$), and OpenFace ($d_{v}=35$) are projected to a unified dimension $d_{h}=128$ via learned linear projections prior to entering any module. The cross-modal alignment module adopts a 4-head attention architecture to facilitate semantic interaction across modalities. The gating MLPs in both the TGA and DGF modules consist of a single linear layer with hidden size equal to $d_{h}=128$, followed by Sigmoid activation; no additional nonlinearity is introduced in order to keep the gates computationally lightweight. The global dropout rate is set to 0.6 for CMU-MOSI and 0.4 for CMU-MOSEI to suppress overfitting.

For the contrastive learning temperature τ, a sensitivity analysis was conducted by varying τ∈{0.05,0.07,0.1,0.2,0.5} on the CMU-MOSEI validation set; τ=0.07 consistently yielded the best performance, as shown in Table 2. The overall training objective comprises three components weighted as *w*_1_ = 1.0, *w*_2_ = 0.1, and *w*_3_ = 0.1 for classification, polarity, and intensity losses respectively, drawing from the experimentally validated scheme in PS-Mixer [40].

**Table 2:** Sensitivity analysis of temperature parameter τ on the CMU-MOSEI dataset.

Method	MAE	Corr	Acc-2(%)	F1(%)
τ=0.05	0.5307	0.7803	84.33	84.07
** τ=0.07 **	**0.5140**	**0.7924**	**84.66**	**84.58**
τ=0.1	0.5198	0.7877	84.21	83.97
τ=0.2	0.5275	0.7802	81.75	81.13
τ=0.5	0.5296	0.7768	83.15	82.71

### 4.4 Baselines

To evaluate the effectiveness of our proposed model, we selected a series of representative baseline methods and state-of-the-art approaches for a fair comparison.

MCAN(2025) [51] addresses inter-modal conflicts in multimodal sentiment analysis by separating and specifically modeling conflicting information from unimodal and bimodal representations.

MCL-MCF(2025) [52] addresses modality heterogeneity in multimodal sentiment analysis through a progressive fusion strategy that combines multi-level contrastive learning and convolutional fusion.

MSAN(2024) [53] proposes a 3D attention-based multimodal sentiment analysis framework that progressively constructs a tri-modal temporal attention mechanism to achieve comprehensive interaction and fusion of sentiments across modalities.

CMHFM(2024) [54] introduces a cross-modal hierarchical fusion approach that enhances multimodal feature representations through multi-task learning across single-, dual-, and tri-modal tasks.

ALMT(2023) [26] introduces an Adaptive Hypermodal Learning (AHL) module that suppresses irrelevant representations from video and audio features under linguistic guidance, thereby achieving multimodal fusion with complementary joint representations.

TETFN(2023) [11] proposes a Text-Enhanced Transformer Fusion Network that integrates textual information to learn sentiment-relevant non-verbal representations through text-based multi-head attention.

PS-Mixer(2023) [40] determines sentiment polarity and intensity by designing Polarity Vectors (PV) and Strength Vectors (SV). It employs an MLP-based communication module to fully fuse multimodal features, ultimately generating a joint fusion vector for sentiment analysis.

self-MM(2021) [15] is a self-supervised multimodal learning framework that generates modality-specific supervision signals from multimodal sentiment labels to learn discriminative unimodal representations.

MISA(2020) [14] enhances multimodal fusion by decomposing each modality’s representation into modality-invariant and modality-specific subspaces, enabling more effective sentiment prediction.

MulT(2019) [10] addresses misalignment and long-range dependency issues in multimodal human language sequences through a directional paired cross-modal attention mechanism.

RoBERTa (Text-only): A unimodal baseline using only textual features extracted from RoBERTa, without incorporating audio or visual modalities. This baseline evaluates the contribution of multimodal fusion beyond the dominant textual modality.

To ensure experimental fairness, all baseline results reported in Tables 3 and 4 were obtained by re-running the original implementations under the same feature protocol (RoBERTa for text, OpenFace for video, COVAREP for audio) and the same train/valid/test splits. For baselines whose original implementations used different feature extractors (e.g., BERT instead of RoBERTa), we replaced the text encoder with RoBERTa and fine-tuned accordingly. Results that could not be reproduced under the same feature setting due to unavailable code are marked with * those cases.

**Table 3:** Performance Comparison on the MOSI Dataset. The left side of the “/” in ACC-2 and F1 Score is calculated as negative/non-negative, while the right side is calculated as negative/positive. The best result is highlighted in bold.

Model	MAE↓	Corr↑	Acc-2(%)↑	F1(%)↑	Acc-7(%)↑
MCAN (2025)^*^	0.6850	0.8001	–/84.5	–/84.8	43.10
MCL-MCF (2025)	0.6900	0.7890	84.81/86.89^†^	84.72/86.88	–
MSAN (2024)^*^	0.7124	0.7941	–/83.68	–/83.71	–
CMHFM (2024)^*^	0.7971	0.7533	81.17/82.71	81.12/82.72	40.17
ALMT (2023)	0.7171^†^	0.7887^†^	83.38^†^/85.67	83.28^†^/85.64	**48.25** ^‡^
TETFN (2023)	0.7425	0.7886	81.63/83.38	81.61/83.42	43.15
PS-Mixer (2023)	0.7940	0.7499	81.34/82.77	81.33/82.71	42.56
self-MM (2021)	0.7180	0.7663	81.44/ 83.46	81.36/83.43	44.67
MISA (2020)	0.7765	0.7781	81.84/83.85	81.82/83.58	41.37
MulT (2019)	0.8799	0.7022	79.71/80.98	79.63/80.95	36.91
RoBERTa-only	0.7921	0.7802	81.48/83.23	81.45/83.14	40.23
**TDGN (ours)**	**0.6830±0.0034**	**0.8030±0.0052**	**85.28±0.26/87.04±0.21**	**85.27±0.38/87.09±0.27**	**48.02±0.27**

^*^ Results reported from original papers. All other results are reproduced under the same configuration.

^†^ p < 0.001 compared to TDGN (paired t-test, two-tailed).

^‡^ Not significant compared to TDGN (p = 0.29).

**Table 4:** Performance Comparison on the MOSEI Dataset. The left side of the “/” in ACC-2 and F1 Score is calculated as negative/non-negative, while the right side is calculated as negative/positive. The best result is highlighted in bold.

Model	MAE↓	Corr↑	Acc-2(%)↑	F1(%)↑	Acc-7(%)↑
MCAN (2025)^*^	0.5273	0.7901	–/85.81	–/85.9	51.60
MCL-MCF (2025)	0.5364	0.7671	84.2/86.4^†^	84.4/86.3	–
MSAN (2024)^*^	0.5210	0.7680	–/83.87	–/83.91	–
CMHFM (2024)^*^	0.5481	0.7373	84.07/84.45	83.85/83.97	52.83
ALMT (2023)	0.5352	0.7730	83.00/85.58	83.21/85.39	53.72
TETFN (2023)	0.5373	0.7696	84.12/86.21	84.35/86.11	53.90
PS-Mixer (2023)	0.5359	0.7669	82.29/85.81	81.81/85.80	52.25
self-MM (2021)	0.5309	0.7649	81.76/ 83.15	81.82/83.90	53.87
MISA (2020)	0.5575	0.7515	80.67/84.67	81.12/84.66	52.05
MulT (2019)	0.5593	0.7331	81.15/84.63	81.56/84.52	52.84
RoBERTa-only	0.5207	0.7833	82.55/84.18	82.09/84.16	53.63
**TDGN (Ours)**	**0.5140±0.0056**	**0.7924±0.0097**	**84.66±0.28/87.05±0.28**	**84.58±0.29/87.05±0.28**	**54.73±0.31**

^*^ Results reported from original papers. All other results are reproduced under the same configuration.

^†^ p < 0.001 compared to TDGN (paired t-test, two-tailed).

As shown in the Tables 3 and 4, TDGN achieves the best overall performance on most evaluation metrics on both the MOSI and MOSEI datasets. These results confirm the effectiveness of the text-guided alignment, dual-layer gated fusion mechanisms and text-anchored contrastive learning in integrating multimodal sentiment information and enhance overall robustness.

In the MOSI and MOSEI datasets, significant disparities exist in the quality and signal-to-noise ratio across different modalities. The MOSI dataset is relatively small, with all data sourced from movie reviews. The text modality exhibits high quality and direct expression, whereas the video and audio modalities, constrained by filming conditions and background interference, often contain substantial noise unrelated to sentiment. Although the MOSEI dataset is larger and more diverse in scenarios, it similarly suffers from modal imbalance. Noise in audiovisual modalities is more complex.

These inter-modal quality disparities make it challenging for traditional averaging fusion or attention mechanisms to adaptively balance modal contributions, rendering them susceptible to interference from low-quality modalities. While TDGN consistently surpasses all baselines in MAE, Corr, Acc-2, and F1 metrics, its Acc-7 performance is slightly lower than that of ALMT. This minor difference is likely due to the hierarchical hypermodality structure in ALMT, which provides finer granularity in discrete-level sentiment classification. Nevertheless, TDGN achieves overall superior performance and generalization ability, particularly on the noise-prone MOSEI dataset, confirming the efficacy of its adaptive gating mechanism and text-guided fusion strategy in complex multimodal environments.

### 4.5 Ablation study

#### 4.5.1 Ablation study on TGA.

We designed a series of ablation experiments to evaluate the contribution of text-dominant cross-modal attention mechanisms in multimodal sentiment analysis. Three distinct architecture variants were tested:(1) Complete removal of cross-modal attention (w/o Cross-Attn), (2) Removal of text-video attention only (w/o T → V Attn), (3) Removal of text-audio attention only (w/o T → A Attn), evaluated on the MOSI and MOSEI datasets.(4) Removal of the entire Text-Anchored Gated Attention module, where the raw modality features are directly passed to the pooling layer and subsequent fusion without text-guided alignment (w/o TGA).

The trends summarized in Tables 5 and 6 reveal the following observations: Completely removing cross-modal attention (w/o Cross-Attn) leads to a significant performance drop: on MOSI, MAE increases from 0.683 to 0.697 and Corr decreases by 0.03; on MOSEI, MAE rises from 0.514 to 0.561. This indicates that cross-modal attention plays a crucial role in capturing semantically complementary information.

**Table 5:** Ablation results for the TGA module on the CMU-MOSI dataset.

Method	MAE	Corr	Acc-2(%)	F1(%)
w/o Cross-Attn	0.6968	0.7701	84.85	84.89
w/o T→V Attn	0.6994	0.7666	80.04	79.50
w/o T→A Attn	0.6987	0.7667	84.85	84.33
w/o TGA	0.7542	0.74755	83.06	82.82
**TDGN**	**0.6830**	**0.8030**	**85.28**	**85.27**

**Table 6:** Ablation results for the TGA module on the CMU-MOSEI dataset.

Method	MAE	Corr	Acc-2(%)	F1(%)
w/o Cross-Attn	0.5612	0.7726	83.45	83.75
w/o T→V Attn	0.5297	0.7791	83.61	83.27
w/o T→A Attn	0.5406	0.7644	82.53	82.12
w/o TGA	0.5250	0.7822	77.86	77.89
**TDGN**	**0.5140**	**0.7924**	**84.66**	**84.58**

Removing text-video attention (w/o T → V Attn) has the most pronounced impact on performance. Specifically, Acc-2 on MOSI drops from 85.28% to 80.04%, and F1 decreases by approximately 6 percentage points. This indicates that the video modality makes a significant contribution to aligning facial expressions and posture cues, while text-video attention effectively suppresses visual noise and enhances semantic consistency.

Removing text-audio attention (w/o T → A Attn) also causes some performance degradation, though to a lesser extent. This indicates that the audio modality’s emotional intensity information is well-modeled under text guidance, but its influence on the final decision is slightly weaker than that of the visual modality.

TDGN achieves optimal performance on both datasets, demonstrating the effectiveness of text-dominated cross-modal attention in modeling semantic alignment and modal complementarity.

In summary, the introduction of cross-modal attention significantly enhances the model’s emotion recognition performance. Specifically, the design using text as a semantic anchor enables efficient feature alignment and information fusion within the multimodal space.

#### 4.5.2 Ablation study on DGF.

To evaluate the effectiveness of the proposed gated fusion mechanism in multimodal sentiment analysis, we designed and conducted ablation experiments on seven model variants:

(1) Simplified-Gate: Simplifies the two-layer gated structure to a single-layer linear weighting, retaining only fixed fusion weights between modalities; (2) w/o Inter-Gate: Removes inter-modal interaction gating while retaining intra-modal gating to validate the necessity of dynamic inter-modal coordination; (3) w/o Intra-Gate: Eliminates modality-specific gating, retaining only interaction gating to test the importance of intra-modal semantic filtering; (4) Concat-MLP: Concatenates features from all modalities and feeds them into an MLP classifier as a no-gating baseline. (5) Weighted: Replaces the dynamic gating mechanism with static learned scalar weights for modality integration; (6) Gated: Applies a standard flat gating mechanism across all modalities without the hierarchical intra- and inter-modal distinction; (7) No-text guide: Removes the text-guided constraints during the fusion phase to verify the necessity of text-anchored semantic references.(8) Late-fusion: Independently encodes each modality and combines their outputs via weighted averaging at the decision level, bypassing any cross-modal interaction or gating during feature learning.

As shown in Tables 7 and 8, TDGN achieves optimal results on both datasets, validating the effectiveness of the dual-gated mechanism in dynamic multimodal fusion.

**Table 7:** Ablation results for different fusion strategies on the CMU-MOSI dataset.

Method	MAE	Corr	Acc-2(%)	F1(%)
Simplified-Gate	0.7301	0.7693	83.09	83.31
w/o Inter-Gate	0.7253	0.7745	82.70	82.65
w/o Intra-Gate	0.7328	0.7644	84.13	84.15
Concat-MLP	0.7451	0.7781	82.29	82.30
Weighted	0.7601	0.7396	81.85	81.52
Gated	0.7406	0.7854	82.65	82.67
No-text guide	0.7722	0.7710	83.21	83.26
Late fusion	0.7925	0.7771	82.79	82.75
**TDGN**	**0.6830**	**0.8030**	**85.28**	**85.27**

**Table 8:** Ablation results for different fusion strategies on the CMU-MOSEI dataset.

Method	MAE	Corr	Acc-2(%)	F1(%)
Simplified-Gate	0.5268	0.7740	82.33	82.67
w/o Inter-Gate	0.5414	0.7654	83.18	83.09
w/o Intra-Gate	0.5364	0.7716	83.91	84.10
Concat-MLP	0.5574	0.7482	80.02	80.06
Weighted	0.5359	0.7773	83.57	83.28
Gated	0.5166	0.7868	83.61	83.52
No-text guide	0.5354	0.7710	82.67	82.39
Late fusion	0.5266	0.7870	81.97	81.41
**TDGN**	**0.5140**	**0.7924**	**84.66**	**84.58**

The simplified gate mechanism (Simplified-Gate) leads to a significant performance drop, indicating that the hierarchical gate design plays a crucial role in modal selection and weight balancing.

Removing the interaction gate (w/o Inter-Gate) degrades model performance on both datasets, demonstrating that inter-modal gating effectively models complementary and dependent relationships between modalities.

Eliminating the modality-specific gate (w/o Intra-Gate) similarly causes performance degradation, indicating that intra-modal adaptive filtering is essential for suppressing redundant features and enhancing primary semantic components.

Simple concatenation (Concat-MLP) serves as the gating-free fusion baseline, exhibiting the most pronounced performance decline, validating the central role of gating structures in modal fusion.

The static weighting approach (Weighted) yields poor results, revealing that fixed modality weights fail to dynamically adapt to the varying signal-to-noise ratios across different samples. Meanwhile, the flat gating baseline (Gated) generally outperforms Concat-MLP but falls short of TDGN, further emphasizing that a hierarchical separation of intra-modal enhancement and inter-modal balancing is superior to a generic, single-stage gating layer. Furthermore, the removal of textual guidance (No-text guide) results in noticeable performance degradation, confirming that relying on text as a semantic anchor is crucial for effectively steering the fusion process. The late-fusion baseline consistently underperforms TDGN across both datasets demonstrating that deferring modality integration to the decision level sacrifices the fine-grained cross-modal interactions and adaptive noise suppression that are essential for robust multimodal sentiment understanding.

In summary, the synergistic interaction between the intra-modal semantic filtering and inter-modal dynamic coordination effectively enhances the discriminative power and robustness of fused features. The hierarchical dual-gated architecture enables the model to demonstrate superior generalization capabilities and interpretability in multimodal sentiment recognition tasks.

#### 4.5.3 Ablation study on TACL.

To evaluate the effectiveness of the proposed text-anchored contrastive learning mechanism in multimodal sentiment analysis, we designed and conducted ablation experiments on five model variants:

(1) w/o Contrastive Loss: Removes the text-anchored contrastive learning constraints, relying solely on classification loss for training. (2) Similarity Only: During fusion, only the similarity features *h*_*sim*_ are retained, while the dissimilarity features *h*_*dis*_ are discarded. (3) Dissimilarity Only: Conversely, only the dissimilarity features *h*_*dis*_ are retained, with similarity features *h*_*sim*_ discarded. (4) Single Positive Pair-video: The contrastive learning framework is constrained to use only text-video positive pairs, excluding text-audio pairs. (5) Single Positive Pair-audio: Similarly, only text-audio positive pairs are retained in contrastive learning, excluding text-video pairs.

TDGN incorporates both similarity and dissimilarity features with text-anchored contrastive learning using all positive pairs (text-video and text-audio). As shown in Tables 9 and 10, the key findings are summarized as follows:

**Table 10:** Ablation results for different components on the CMU-MOSEI dataset.

Method	MAE	Corr	Acc-2(%)	F1(%)
w/o Contrastive Loss	0.5356	0.7673	82.50	82.30
Similarity Only	0.5545	0.7612	83.66	83.40
Dissimilarity Only	0.5575	0.7542	83.32	82.92
Single Positive-Pair-video	0.5280	0.7808	83.51	83.12
Single Positive-Pair-audio	0.5307	0.7885	83.06	82.63
**TDGN**	**0.5140**	**0.7924**	**84.66**	**84.58**

**Table 9:** Ablation results for different components on the CMU-MOSI dataset.

Method	MAE	Corr	Acc-2(%)	F1(%)
w/o Contrastive Loss	0.7072	0.7732	83.10	83.00
Similarity Only	1.0140	0.6291	75.80	75.82
Dissimilarity Only	0.9856	0.6667	76.82	76.81
Single Positive Pair-video	1.0283	0.6482	74.78	74.79
Single Positive Pair-audio	0.9291	0.6977	79.44	79.51
**TDGN**	**0.6830**	**0.8030**	**85.28**	**85.27**

Removing the contrastive loss (w/o Contrastive Loss) leads to a performance decline. This confirms that implicit fusion alone cannot resolve semantic misalignment. TACL effectively constrains the cross-modal latent space by using text as a stable anchor to enforce semantic consistency and suppress multimodal noise.

Furthermore, We compared TDGN against variants using only similarity (Similarity Only) or dissimilarity (Dissimilarity Only) components. The drastic Acc-2 drop from 85.28% to 75.80% in the Similarity Only variant indicates that discarding modality-specific cues significantly weakens the model. Conversely, the Dissimilarity Only variant lacks the necessary shared semantic consensus. The superiority of the Full-model proves that orthogonal decoupling preserves complementary modality-specific information while maintaining global semantic alignment.

Restricting contrastive targets to a single modality (Single Positive Pair-video/audio) results in suboptimal performance. This suggests that single-pair alignment is prone to biased optimization and noise from unreliable modalities. By simultaneously anchoring both audio and video to text, TACL establishes a robust semantic consensus that enhances the stability and discriminative power of fused representations.

In conclusion, these results demonstrate that the text-anchored asymmetric contrastive strategy and feature decoupling are vital to the robustness of the TDGN framework.

### 4.6 Visualization

To gain deeper insights into the effectiveness and interpretability of each module within TDGN, this section conducts a series of visualization experiments analyzing aspects such as feature alignment, feature fusion, contrastive learning optimization, and gated activation distributions. The results provide intuitive validation of the model design’s rationality and the semantic consistency of multimodal sentiment representations.

**Fig 5:**
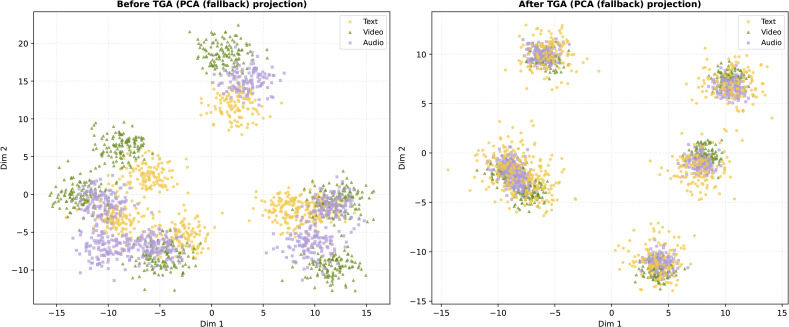
t-SNE visualization of multimodal representations before and after applying the TGA module. Left: the features are misaligned and distributed irregularly across modalities. Right: after TGA, the features exhibit improved alignment and compact clustering across text, video, and audio modalities.

Fig 5 illustrates the t-SNE visualization of multimodal feature distributions before and after applying the TGA module. As shown in the left subfigure, the representations of text, video, and audio modalities are scattered and exhibit weak inter-modal correspondence, indicating poor alignment in the latent space. After introducing the TGA module (right subfigure), the modality-specific features become tightly clustered, with samples from different modalities corresponding to the same semantic class being well-aligned. This result demonstrates that TGA effectively enhances semantic consistency across modalities, yielding a more coherent and discriminative shared representation space.

**Fig 6:**
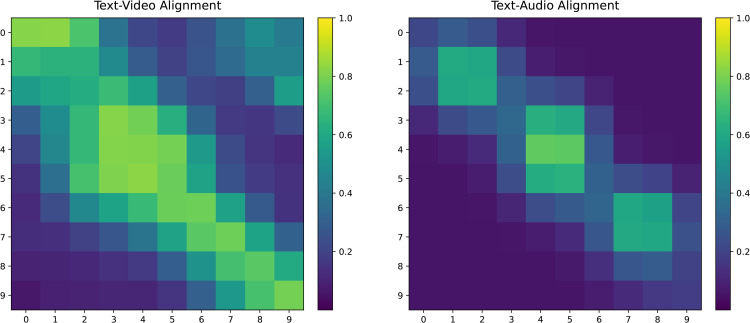
Cross-modal attention heatmaps reveal distinct alignment patterns between modalities. The Text–Video map (left) exhibits a prominent near-diagonal activation pattern, indicating strong fine-grained temporal correspondence between textual tokens and video frames. The Text–Audio map (right) displays sparse, block-structured activations concentrated at a few emotionally salient speech intervals, reflecting selective attention to affectively relevant acoustic segments while suppressing redundant acoustic information. Brighter regions (higher attention weight) indicate stronger cross-modal correspondence. These contrasting patterns demonstrate the efficacy of the text-anchored cross-modal attention mechanism in achieving modality-specific alignment under a unified semantic guidance framework.

Fig 6 illustrates the cross-modal attention heatmaps for Text–Video and Text–Audio modalities. For Text–Video alignment (Fig 6, left), the attention map displays a pronounced near-diagonal activation pattern, indicating that textual tokens consistently attend to temporally adjacent video frames. This structured diagonal distribution demonstrates that the TGA module successfully captures fine-grained temporal correspondences between language and visual sequences. The text modality, acting as the semantic query, directs attention toward video frames that carry visually consistent emotional cues, effectively suppressing irrelevant frame interference and yielding a stable, sequential alignment.

In contrast, the Text–Audio alignment map (Fig 6, right) exhibits a sparser and more localized activation structure. Rather than a continuous diagonal, attention weights concentrate in a small number of discrete high-intensity blocks, corresponding to emotionally salient speech intervals. This pattern indicates that the model selectively focuses on acoustically informative segments-such as prosodically prominent regions-while attenuating redundant or noisy acoustic frames. The block-like structure further reflects the temporal granularity difference between linguistic tokens and acoustic frames, and confirms that text-guided attention effectively filters audio content according to semantic relevance.

Collectively, these heatmaps confirm the effectiveness of the text-anchored cross-modal attention mechanism in achieving stable multimodal alignment. Text, as a semantically dense modality, guides video features toward stable, consistent alignment while directing the model’s focus toward semantically critical regions in audio. This process yields more refined and discriminative representations, providing a cleaner and more informative foundation for subsequent gated fusion and contrastive learning stages.

**Fig 7:**
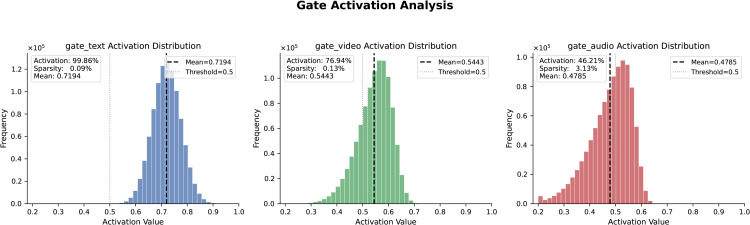
Distribution of gated activation values for three modalities during fusion. Histograms show Sigmoid gate activations for text, video, and audio, with a threshold of 0.5. Text exhibits a highly concentrated high-activation distribution, indicating near-complete feature preservation. Video shows a moderately dispersed, slightly left-skewed distribution, reflecting selective filtering. Audio presents the broadest and most left-skewed distribution, indicating substantial suppression. These patterns demonstrate modality-aware gating aligned with heterogeneous noise levels.

Fig 7 illustrates the modality-specific gate activation distributions during DGF fusion, revealing clear asymmetry aligned with heterogeneous signal quality. The text gate is sharply concentrated above the activation threshold, indicating that textual features are almost fully preserved. In contrast, the video gate exhibits a broader, mildly left-skewed distribution, suggesting moderate suppression of redundant visual information. The audio gate shows the widest and most left-skewed distribution, with nearly half of its dimensions suppressed, consistent with higher acoustic noise. This progressive reduction (text > video > audio) provides interpretable evidence that DGF performs noise-aware, modality-differentiated feature filtering, rather than uniform fusion, thereby validating the design of TDGN’s asymmetric gating mechanism.

## 5 Discussion

In this study, we presented the Text-Guided Dual-Gated Network (TDGN) for multimodal sentiment analysis, designed to address challenges like semantic alignment, modality imbalance, and noise interference across text, audio, and visual data. Our experiments on the CMU-MOSI and CMU-MOSEI benchmarks demonstrated TDGN’s superiority, achieving state-of-the-art results in accuracy, robustness, and generalization.

A key innovation of TDGN is the Text-Anchored Gated Attention (TGA) module, which uses text as a semantic anchor to guide alignment between audio and visual modalities. Specifically, TGA operates at the alignment stage by applying pre-attention gating to suppress unreliable audiovisual channels before cross-modal attention is computed, thereby preventing noisy signals from propagating into the alignment process. This approach proved effective in mitigating noise from non-verbal modalities and emphasized emotionally relevant features. Experimental results show that TGA significantly improved alignment, reflected in reduced MAE and enhanced Corr, Acc-2, and F1 scores.

Another major contribution is the Dual-layer Gated Fusion (DGF) module, which employs both intra- and inter-modal gating mechanisms to balance the contributions of each modality. Unlike TGA, which targets noise suppression prior to alignment, DGF operates downstream at the fusion stage on already-aligned representations: intra-modal gating enhances discriminative modality-specific features, while inter-modal gating dynamically reweights cross-modal contributions to achieve global semantic balance. This stage-wise separation ensures that noise filtering and modality balancing are addressed independently, enabling more interpretable error correction across the pipeline. This dynamic fusion prevents the over-assimilation of text features while preserving the unique characteristics of audio and visual data. Ablation studies validated the importance of these gating strategies, as removing either intra- or inter-modal gating led to significant performance degradation.

Additionally, Text-Anchored Contrastive Learning (TACL) was integrated to enforce consistency across modalities. TACL not only enhanced model stability and semantic coherence but also ensured high-quality fusion despite the heterogeneous nature of the input data.

Despite these advancements, the current framework also has several limitations. First, its effectiveness depends on the reliability of textual semantics; when the text is ambiguous, sarcastic, or weakly emotional, text anchoring may become less reliable. Second, the model relies on pre-extracted OpenFace and COVAREP features, so performance is partly affected by external feature protocols. Third, the present study focuses on complete-modality settings and does not explicitly address missing-modality cases.

While TDGN outperforms many state-of-the-art models in robustness, its slightly lower Acc-7 score compared to ALMT suggests room for improvement in fine-grained sentiment classification. Future models could explore hierarchical fusion techniques or hypermodal learning strategies to enhance sentiment categorization at more granular levels.

In conclusion, TDGN offers an effective solution for multimodal sentiment analysis, tackling key challenges of alignment and modality imbalance. Its use of text-guided attention, gated fusion, and contrastive learning enables superior performance while maintaining interpretability and adaptability. This work sets the stage for future research focused on improving fusion techniques and exploring joint feature extraction methods for multimodal sentiment analysis.

## 6 Conclusion

This paper proposes TDGN designed to address the key challenges of semantic inconsistency, modality imbalance, and noise interference in multimodal sentiment analysis. By employing TGA module, TDGN leverages textual semantics as a stable reference to achieve fine-grained alignment between audio and visual modalities, effectively suppressing redundant and noisy signals. Furthermore, the DGF mechanism jointly models intra-modal saliency and inter-modal complementarity, enabling adaptive and balanced multimodal integration. To preserve the diversity of nonverbal information, TACL strategy is introduced to enhance semantic consistency across modalities while maintaining discriminative modality-specific cues.

Comprehensive experiments on CMU-MOSI and CMU-MOSEI demonstrate that TDGN achieves state-of-the-art performance across multiple evaluation metrics, confirming the effectiveness of the proposed alignment, fusion, and contrastive components. Ablation and visualization analyses further verify the interpretability and robustness of the dual-gated architecture, revealing how text-guided alignment improves semantic coherence and adaptive fusion enhances model stability under noisy conditions.

Despite its promising results, TDGN still relies on pre-extracted static audio–visual features, which may limit its ability to capture subtle temporal variations such as micro-expressions and prosodic dynamics. In future work, we plan to develop end-to-end multimodal encoders that jointly optimize feature extraction and fusion, and explore noise-aware and adaptive fusion mechanisms to improve the model’s scalability and real-world applicability in dynamic affective scenarios.
